# Supplementary material for: CKAMP44 modulates integration of visual inputs in the lateral geniculate nucleus
Source: Nat Commun. 2018 Jan 17;9:261. doi: 10.1038/s41467-017-02415-1 (PMC5772470; doi:10.1038/s41467-017-02415-1)
Supplement: Supplementary file 1 — Supplementary Information [file 41467_2017_2415_MOESM1_ESM.pdf]

Supplementary Figure 1

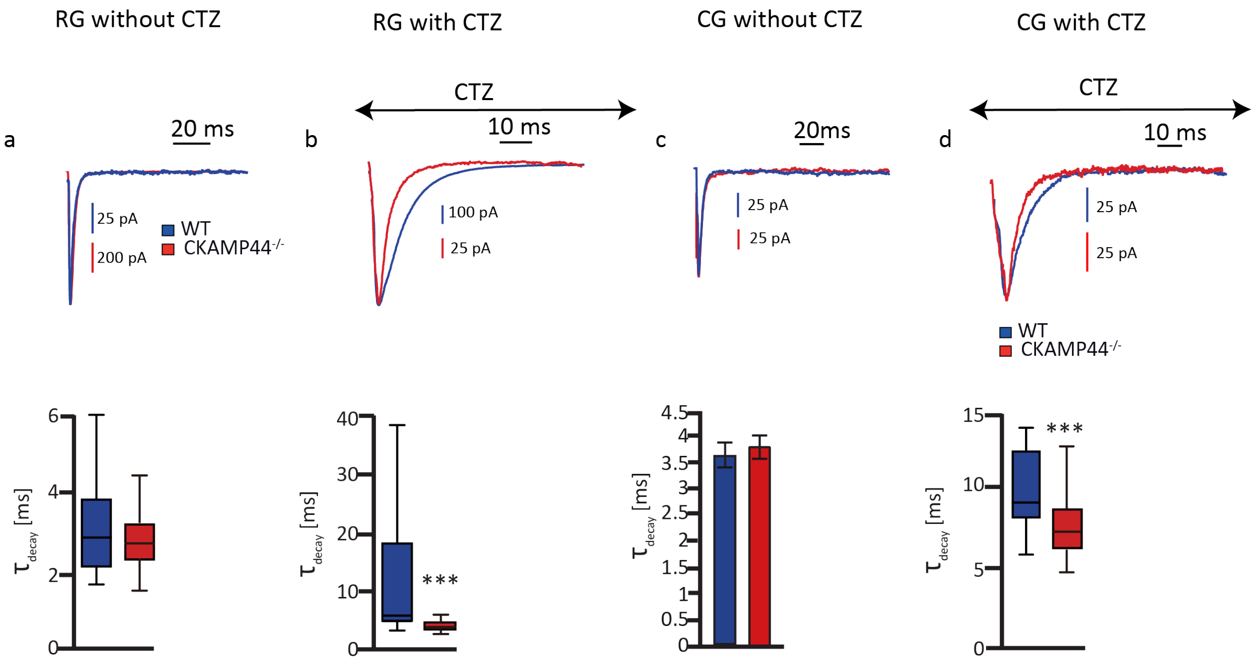

Supplementary Figure 1 In the presence of cyclothiazide (CTZ), CKAMP44 increases τ_{deact} of AMPARs mediated currents.

τ_{deact} of AMPAR-mediated currents in retinogeniculate (a, n= 24 for relay neurons of wildtype and 24 for relay neurons of *CKAMP44*$^{-/-}$ mice; b, n= 21 for relay neuron of wildtype and 24 for relay neurons of *CKAMP44*$^{-/-}$ mice) or corticogeniculate synapses (c, n= 20 for relay neurons of wildtype and 20 for relay neurons of *CKAMP44*$^{-/-}$ mice; d, n= 20 for relay neurons of wildtype and 24 for relay neurons of *CKAMP44*$^{-/-}$ mice) is reduced in CKAMP44$^{-/-}$ mice in the presence, but not in the absence of CTZ. (Box-and-whisker Tukey plots; Mann-Whitney test; except for τ_{deac} in corticogeniculate synapses in the absence of CTZ= mean ± SEM and t-test). Example traces are shown on the top. * = $p < 0.05$, ** = $p < 0.01$, *** = $p < 0.001$.

Supplementary Figure 2

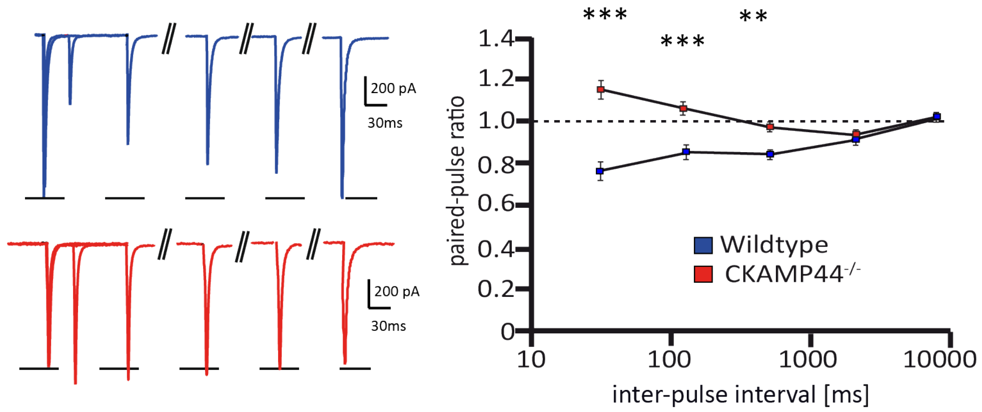

Supplementary Figure 2 Deletion of CKAMP44 increases PPRs of retinogeniculate synapses when using a K^{+}-containing intracellular solution.

PPRs are significantly different between genotypes for inter-pulse intervals of 30, 100 and 300 ms (n= 25 for relay neurons of wildtype and 28 for relay neurons of *CKAMP44*$^{-/-}$ mice). During these PPR recordings, a K^{+}-containing intracellular solution was used in contrast to the Cs^{+}-containing intracellular solution during PPR recordings shown in figure 2. Example traces are shown on the left. (mean ± SEM; PPR with 300ms and 1000ms inter-stimulus interval: Mann-Whitney test, PPR with 10, 30, 100, and 3000ms: t-test). * = $p < 0.05$, ** = $p < 0.01$, *** = $p < 0.001$.

Supplementary Figure 3

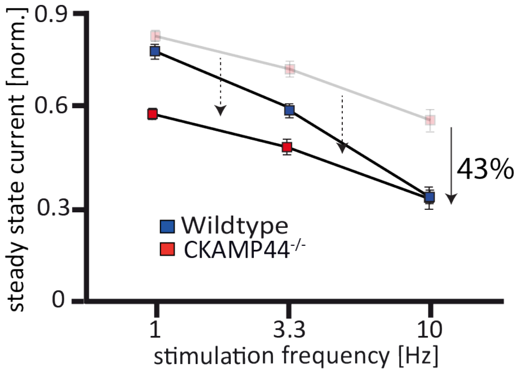

Supplementary Figure 3 CKAMP44 reduces steady-state current amplitude of retinogenicluate synapses at continuous 1 and 3.3 Hz stimulation.

The graph shows the same data as in Figure 3(c), but with a 43 % reduced amplitude of the CKAMP44$^{-/-}$ steady-state values. The graph was adjusted to get an estimate of the steady-state synaptic strength in CKAMP44$^{-/-}$ mice when taking the reduction of AMPA/NMDA ratio into account. (i.e. the reduced synaptic strength due to reduced number of synaptic AMPARs). The graph suggests that the steady-state current amplitude is reduced in CKAMP44$^{-/-}$ mice if RGCs fire with relatively low frequency (1 and 3.3 Hz), but is comparable between genotypes with continuous 10 Hz firing of RGCs. mean ± SEM. * = $p < 0.05$, ** = $p < 0.01$, *** = $p < 0.001$.

Supplementary Figure 4

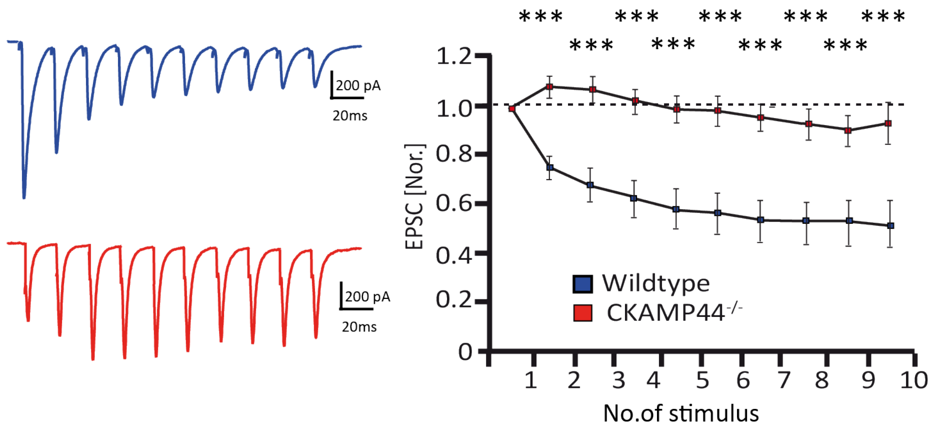

Supplementary Figure 4 Deletion of CKAMP44 increases EPSC amplitudes during a 50 Hz stimulus train.

EPSC amplitudes were significantly bigger in relay neurons of CKAMP44$^{-/-}$ than in those of wildtype mice (n= 25 for relay neurons of wildtype and 27 for relay neurons of *CKAMP44*$^{-/-}$ mice). EPSCs were evoked by stimulating the optic tract with 10 stimuli at 50 Hz. The intracellular solution was the same K^+-containing solution used for current-clamp experiments. EPSC amplitudes were normalized to the amplitude of the first EPSC. Example traces are shown on the left. (mean ± SEM; second, third, fourth and fifth normalized EPSC amplitude: t-test, other amplitudes: Mann-Whitney test). * = $p < 0.05$, ** = $p < 0.01$, *** = $p < 0.001$.

Supplementary Figure 5

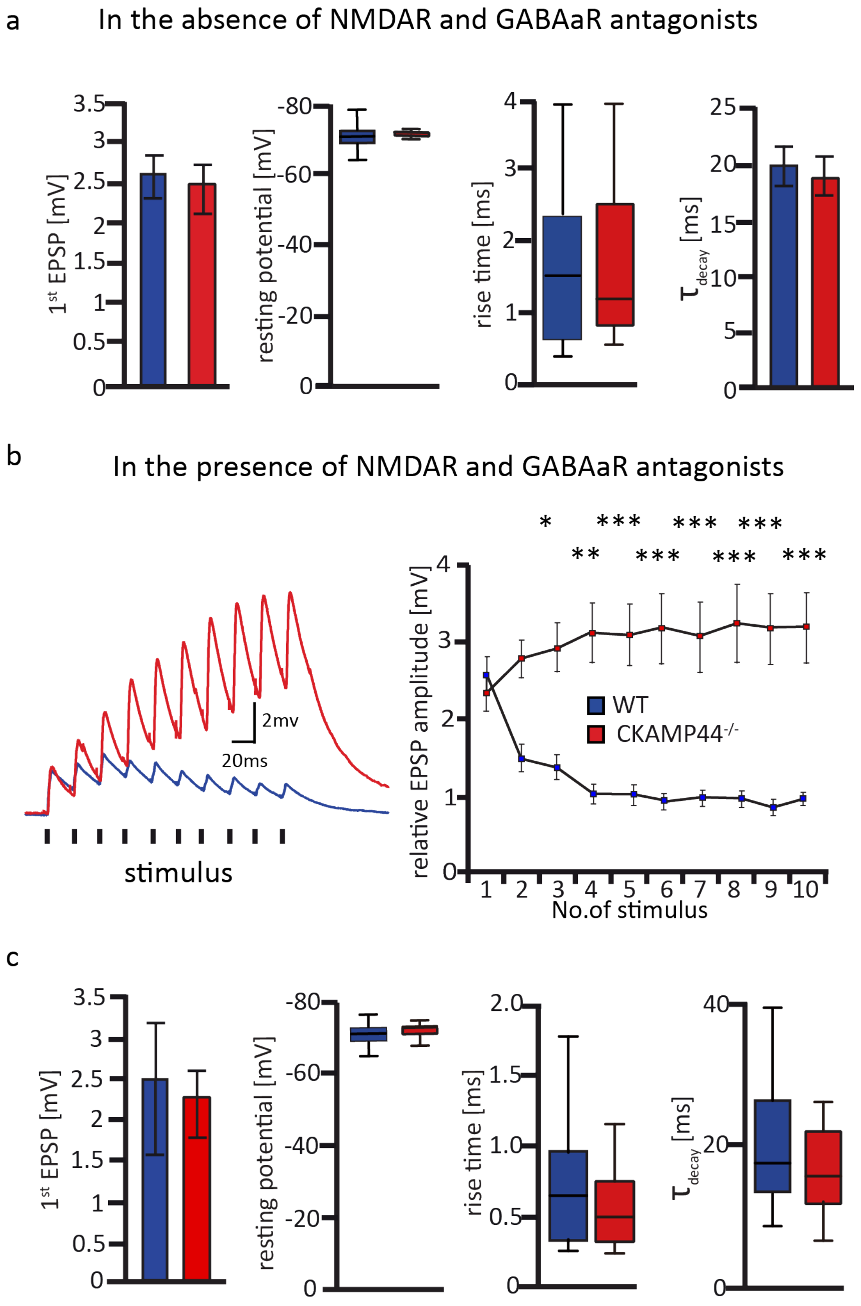

Supplementary Figure 5 Deletion of CKAMP44 increases EPSP amplitudes also in the presence of NMDAR- and $GABA_A$R-antagonists.

(a) The change in EPSP amplitudes shown in Figure 3(a) does not result from differences in 1st EPSP amplitude (n= 18 for relay neurons of wildtype and 22 for relay neurons of *CKAMP44*$^{-/-}$ mice), rise time (n= 22 for relay neurons of wildtype and 21 for relay neurons of *CKAMP44*$^{-/-}$ mice), τ_{decay} (n= 20 for relay neurons of wildtype and 22 for relay neurons of *CKAMP44*$^{-/-}$ mice) or resting potential (n= 21 for relay neurons of wildtype and 19 for relay neurons of *CKAMP44*$^{-/-}$ mice) of relay neurons. (Amplitude and τ_{decay}: mean ± SEM, rise time: Box-and-whisker Tukey plots; amplitude: t-test, τ_{decay} and rise time: Mann-Whitney test). (b) There is a significant difference in EPSP amplitudes in retinogeniculate synapses of wildtype and CKAMP44$^{-/-}$ mice also in the presence of the NMDAR- and $GABA_A$R-antagonists APV and gabazine (n= 20 for relay neurons of wildtype and 20 for relay neurons of *CKAMP44*$^{-/-}$ mice). Example traces are shown on the left. mean ± SEM. (c) Also in the presence of APV and gabazine, there was no difference between genotypes in 1st EPSP amplitude, rise time or τ_{decay}, or resting potential of relay neurons (n= 20 for relay neurons of wildtype and 20 for relay neurons of *CKAMP44*$^{-/-}$ mice; amplitude: mean ± SEM; t-test; rise time, resting potential and τ_{decay}: Box-and-whisker Tukey plots; Mann-Whitney test). * = $p < 0.05$, ** = $p < 0.01$, *** = $p < 0.001$.

Supplementary Figure 6

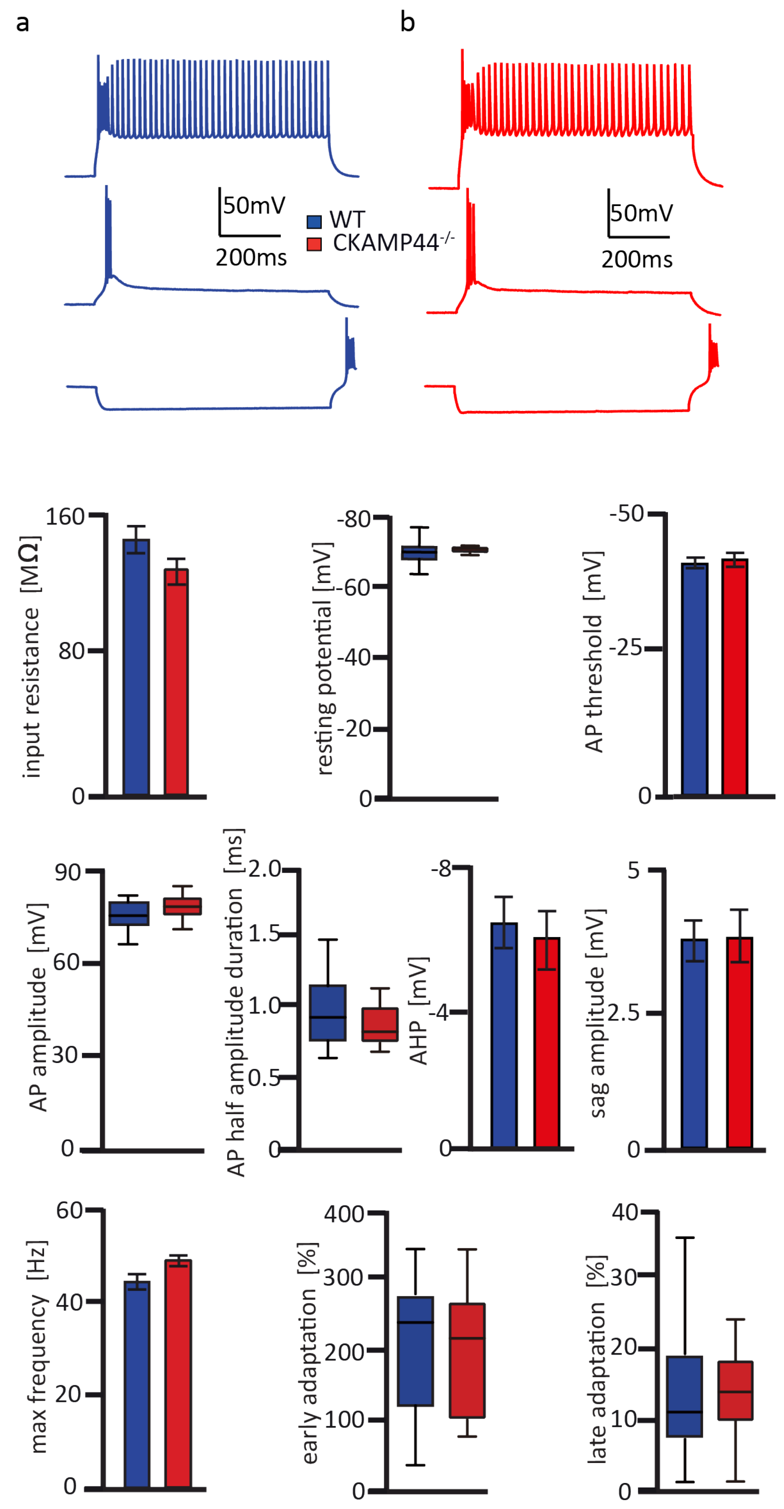

Supplementary Figure 6 Passive and active electrophysiological properties of dLGN neurons are similar in wildtype and CKAMP44$^{-/-}$ mice.

(a, b) Sample traces of the responses of dLGN neurons of wildtype (a) and CKAMP44$^{-/-}$ (b) mice to hyperpolarizing and depolarizing current injections. There is no significant difference in input resistance (mean ± SEM, t-test), resting potential (Box-and-whisker Tukey plots, Mann-Whitney test), sag amplitude (mean ± SEM, t-test), threshold potential (mean ± SEM, t-test), action potential amplitude (Box-and-whisker Tukey plots, Mann-Whitney test), action potential half amplitude duration (mean ± SEM, t-test), after hyperpolarization (AHP) amplitude (mean ± SEM, t-test), max firing frequency (mean ± SEM, t-test), early adaptation (Box-and-whisker Tukey plots, Mann-Whitney test) and, late adaptation (Box-and-whisker Tukey plots, Mann-Whitney test). (n= 21 for relay neurons of wildtype and 19 for relay neurons of *CKAMP44*$^{-/-}$ mice). * = $p < 0.05$, ** = $p < 0.01$, *** = $p < 0.001$.

Supplementary Figure 7

Paired-pulse ratio of retinogeniculate synapses in P60 mice

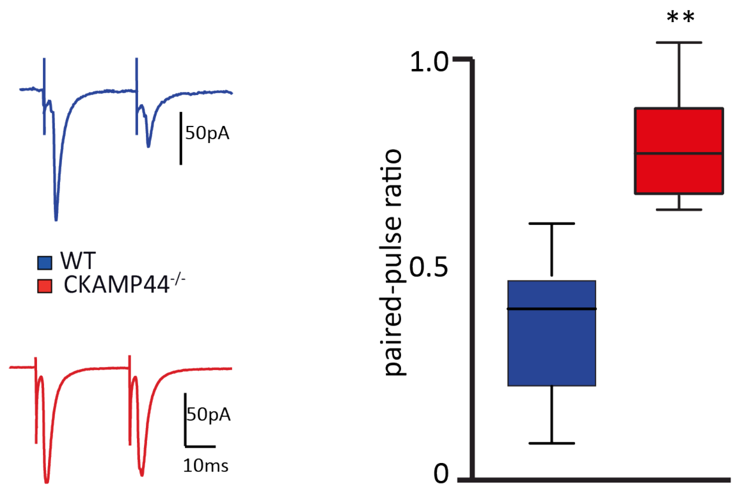

Supplementary Figure 7 CKAMP44 influences AMPAR-mediated currents also in adult mice. There is a significant increase in the paired pulse ratio in retinogeniculate synapses of P60 CKAMP44$^{-/-}$ mice that is comparable to the change observed in younger mice (see Figure 2, n= 12 for relay neurons of wildtype and 9 for relay neurons of *CKAMP44*$^{-/-}$ mice; Box-and-whisker Tukey plots; Mann-Whitney test). Example traces are shown on the left. * = $p < 0.05$, ** = $p < 0.01$, *** = $p < 0.001$.

Supplementary Figure 8

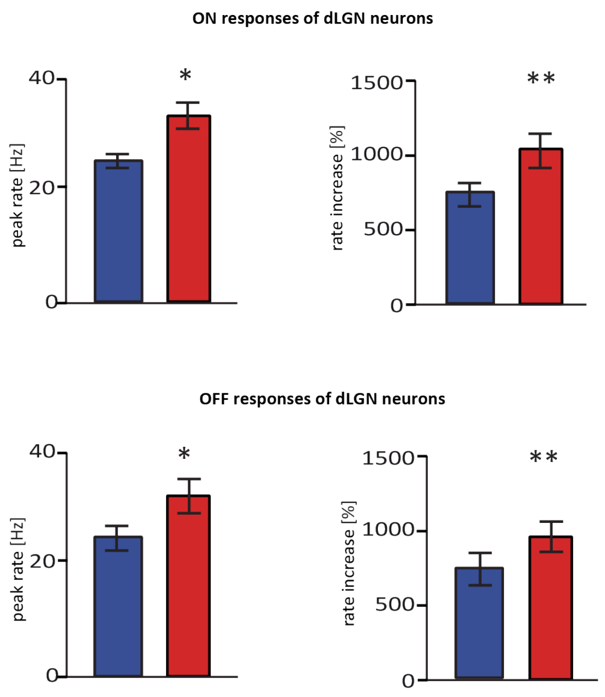

Supplementary Figure 8 Deletion of CKAM P44 increases firing rate and rate increase of dLGN neurons in response to ON- and OFF-stimuli.

Quantifications of firing rates and rate increases from figure 5c shown here as mean ± SEM (ON-response, n= 542 dLGN neurons in 6 wildtype and 430 dLGN neurons in 6 *CKAMP44*$^{-/-}$ mice; OFF-response, n= 309 dLGN neurons in 6 wildtype and 285 dLGN neurons in 6 *CKAMP44*$^{-/-}$ mice). * = $p < 0.05$, ** = $p < 0.01$, *** = $p < 0.001$.

Supplementary Figure 9

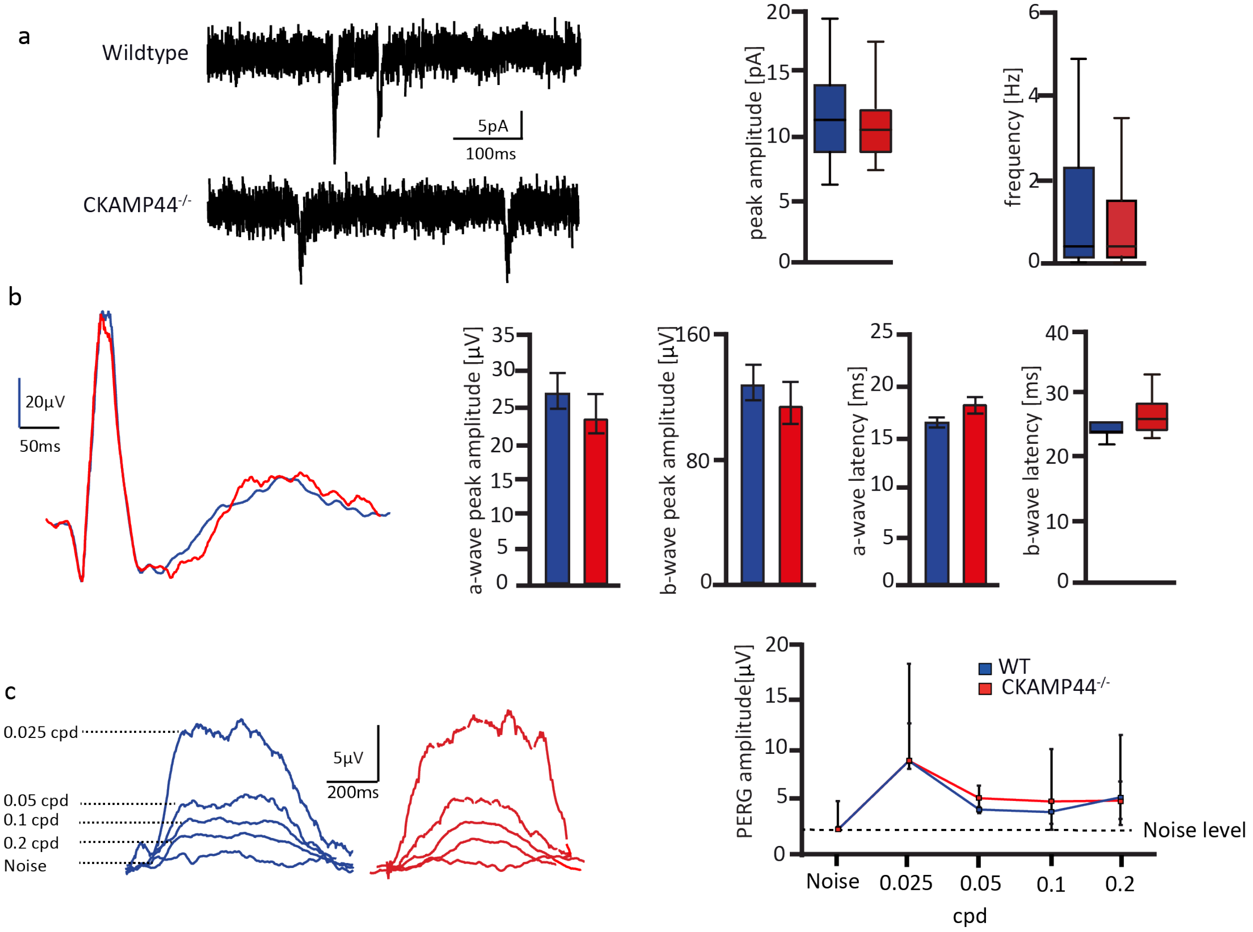

Supplementary Figure 9 AMPAR-mediated mEPSCs of RGCs and retina responses are unaltered in CKAMP44$^{-/-}$ mice.

(a) mEPSC amplitude and frequency is not changed in RGCs of CKAMP44$^{-/-}$ mice (n= 24 for RGCs of wildtype and 29 for RGCs of *CKAMP44*$^{-/-}$ mice; box-and-whisker Tukey plots; Mann-Whitney test). (b and c) Retina responses to flash (b) and drifting pattern (c) stimulation are not significantly changed in CKAMP44$^{-/-}$ mice as evidenced by unaltered a- and b-wave amplitude and latency (b, n= 12 eyes in 6 wildtype mice and 12 eyes in 6 *CKAMP44*$^{-/-}$ mice) and PERG amplitude (c, n= 6 for wildtype and 6 for *CKAMP44*$^{-/-}$ mice) in electroretinogram (ERG) recordings. Drifting pattern ERG was analyzed with spatial frequencies of 0.025 cpd, 0.05 cpd, 0.1 cpd and 0.2 cpd. (a-wave amplitude, latency and b-wave amplitude: mean ± SEM, t-test; b-wave latency and pERG

amplitude: box-and-whisker Tukey plots, Mann-Whitney test). * = $p < 0.05$, ** = $p < 0.01$, *** = $p < 0.001$.

Supplementary Table 1 AMPA/NMDA ratios (see Fig.1c and d)

	WT	N	CKAMP44$^{-/-}$	N
**Retinogeniculate synapses** (median ± IQR, MWW)	4.81 [3.31-8.44]	19	2.75 [1.99-4.04]**	19
**Corticogeniculate synapses** (median ± IQR, MWW)	3.95 [3.02-5.06]	15	1.93 [1.46-2.53]**	18

MWW = Mann-Whitney-Wilcoxon Test

Supplementary Table 2. τ_{decay} in retinogeniculate (RG) and corticogeniculate (CG) synapses (see Supplementary Fig. 1)

	**In the absence of CTZ**					**In the presence of CTZ**			
	WT	N	CKAMP44$^{-/-}$	N		WT	N	CKAMP44$^{-/-}$	N
RG τ_{decay} (ms) (median ± IQR, MWW)	2.87 [2.19-3.81]	24	2.72 [2.38-3.22]	24	RG τ_{decay} (ms) (median ± IQR, MWW)	6.04 [5.34-16.77]	21	4.36 [3.55-4.99]***	24
CG τ_{decay} (ms) (mean ± SEM, STT)	3.63 ± 0.26	20	3.86 ± 0.27	20	CG τ_{decay} (ms) (median ± IQR, MWW)	9.13 [8.33-12.22]	20	7.3 [6.12-8.77]***	24

STT= Student's t-test; MWW = Mann-Whitney-Wilcoxon Test

Supplementary Table 3 Extrasynaptic AMPAR-mediated currents (see Fig.1 f and g)

	WT	N	CKAMP44$^{-/-}$	N
Current amplitude (pA) (median ± IQR, MWW)	31.48 [22.15-45.51]	18	16.03 [11.82-36.68]*	21
10-90% rise time (ms) (median ± IQR, MWW)	0.71 [0.62-0.73]	18	0.77 [0.68-0.93]	21
τ_{deact} (ms) (median ± IQR, MWW)	1.34 [0.97-1.9]	18	1.08 [0.87-1.28]	21
τ_{des} (ms) (mean ± SEM, STT)	2.07 ± 0.199	20	2.08 ± 0.179	17
SS current amplitude (% of peak current) (median ± IQR, MWW)	0.41 [0.17-0.71]	20	0.94 [0.41-1.66]*	17
2nd EPSC amplitude (% of 1st EPSC amplitude) (mean ± SEM, STT)	35.33 ± 3.41	20	72.28 ± 4.1***	17
$\tau_{recovery}$ (ms) (median ± IQR, MWW)	322.85 [195.28-443.78]	20	65.71 [43.5-104.83]***	17

STT= Student's t-test; MWW = Mann-Whitney-Wilcoxon Test

Supplementary Table 4. Paired pulse ratios RG synapses (Cs^+-containing intracellular solution) (see Fig.2a and b)

	Retinogeniculate synapses					Corticogeniculate synapses			
	WT	N	CKAMP44$^{-/-}$	N		WT	N	CKAMP44$^{-/-}$	N
PPR (30ms) (mean ± SEM, MWW)	0.51 ± 0.047	20	0.89 ± 0.085***	18	PPR (30ms) (mean ± SEM, MWW)	1.91 ± 0.143	25	2.21 ± 0.189	22
PPR (100ms) (mean ± SEM, MWW)	0.59 ± 0.031	20	0.93 ± 0.071***	18	PPR (100ms) (mean ± SEM, MWW)	1.71 ± 0.106	25	1.82 ± 0.125	22
PPR (300ms) (mean ± SEM, STT)	0.69 ± 0.026	20	0.92 ± 0.032***	18	PPR (300ms) (mean ± SEM, STT)	1.24 ± 0.053	25	1.37 ± 0.056	22
PPR (1000ms) (mean ± SEM, MWW)	0.82 ± 0.017	20	0.99 ± 0.056**	18	PPR (1000ms) (mean ± SEM, MWW)	1.05 ± 0.026	25	1.06 ± 0.018	22
PPR (3000ms) (mean ± SEM, MWW)	0.99 ± 0.032	20	1.03 ± 0.027	18	PPR (30000ms) (mean ± SEM, STT)	1 ± 0.019	25	0.98 ± 0.017	22

STT= Student's t-test; MWW = Mann-Whitney-Wilcoxon Test

Supplementary Table 5. Paired pulse ratios of RG synapses (K^+-containing intracellular solution) (see Supplementary Fig. 2)

	Retinogeniculate synapses			
	WT	N	CKAMP44$^{-/-}$	N
PPR (30ms inter-stimulus interval) (mean ± SEM, STT)	0.78 ± 0.047	25	1.15 ± 0.059***	28
PPR (100ms inter-stimulus interval) (mean ± SEM, STT)	0.85 ± 0.032	25	1.06 ± 0.033***	28
PPR (300ms inter-stimulus interval) (mean ± SEM, MWW)	0.85 ± 0.023	25	0.97 ± 0.032**	28
PPR (1000ms inter-stimulus interval) (mean ± SEM, MWW)	0.891± 0.03	25	0.93 ± 0.015	28
PPR (3000ms inter-stimulus interval) (mean ± SEM, STT)	1.02 ± 0.013	25	1.01 ± 0.015	28

STT= Student's t-test; MWW = Mann-Whitney-Wilcoxon Test

Supplementary Table 6. Steady state EPSC amplitudes (see Fig. 2c)

	WT	N	CKAMP44$^{-/-}$	N
steady state EPSC [nor.] (1 Hz) (mean ± SEM, MWW)	0.78 ± 0.028	25	0.83 ± 0.027	22
steady state EPSC [nor.] (3.3 Hz) (mean ± SEM, STT)	0.6 ± 0.023	31	0.73 ± 0.027***	24
steady state EPSC [nor.] (10 Hz) (mean ± SEM, STT)	0.33 ± 0.022	33	0.57 ± 0.03***	27

STT= Student's t-test; MWW = Mann-Whitney-Wilcoxon Test

Supplementary Table 7. EPSP amplitudes during 50 Hz train stimulation (see Fig. 3a)

	In the absence of NMDAR and $GABA_AR$ antagonists					In the presence of NMDAR and $GABA_AR$ antagonists			
	WT	N	CKAMP44$^{-/-}$	N		WT	N	CKAMP44$^{-/-}$	N
EPSP 1 (mV) (mean ± SEM, STT)	2.64 ± 0.3	18	2.5 ± 0.28	22	EPSP 1 (mV) (mean ± SEM, STT)	2.51 ±0.25	20	2.31 ± 0.22	20
EPSP 2 (mV) (mean ± SEM, STT)	1.53 ± 0.23	18	2.6± 0.3**	22	EPSP 2 (mV) (mean ± SEM, STT)	1.3 ±0.17	20	2.71 ± 0.26	20
EPSP 3 (mV) (mean ± SEM, MWW)	1.14 ± 0.18	18	2.63 ± 0.33***	22	EPSP 3 (mV) (mean ± SEM, MWW)	1.17 ±0.16	20	2.86 ± 0.33*	20
EPSP 4 (mV) (mean ± SEM, MWW)	1.04 ± 0.21	18	2.85 ± 0.38***	22	EPSP 4 (mV) (mean ± SEM, MWW)	0.86 ±0.14	20	3.08 ±0.4**	20
EPSP 5 (mV) (mean ± SEM, MWW)	0.94 ± 0.18	18	2.79 ± 0.41***	22	EPSP 5 (mV) (mean ± SEM, MWW)	0.81 ±0.12	20	3.06 ±0.44***	20
EPSP 6 (mV) (mean ± SEM, MWW)	0.79 ± 0.16	18	2.9 ± 0.44***	22	EPSP 6 (mV) (mean ± SEM, MWW)	0.72 ±0.09	20	3.14 ±0.48***	20
EPSP 7 (mV) (mean ± SEM, MWW)	0.72 ± 0.14	18	2.93 ± 0.46***	22	EPSP 7 (mV) (mean ± SEM, MWW)	0.77 ±0.11	20	3.06 ±0.5***	20
EPSP 8 (mV) (mean ± SEM, MWW)	0.79 ± 0.16	18	2.85 ± 0.49***	22	EPSP 8 (mV) (mean ± SEM, MWW)	0.74 ±0.11	20	3.17 ±0.55***	20
EPSP 9 (mV) (mean ± SEM, MWW)	0.7 ± 0.13	18	2.82 ± 0.51***	22	EPSP 9 (mV) (mean ± SEM, MWW)	0.65 ±0.1	20	3.15 ±0.53***	20
EPSP 10 (mV) (mean ± SEM, MWW)	0.72 ± 0.14	18	3.05 ± 0.54***	22	EPSP 10 (mV) (mean ± SEM, MWW)	0.68 ±0.09	20	3.15 ±0.51***	20

STT= Student's t-test; MWW = Mann-Whitney-Wilcoxon Test

Supplementary Table 8. Normalized EPSC amplitudes with 50 Hz stimulation (K^+-containing intracellular solution) (see Supplementary Fig. 4)

	WT	N	CKAMP44$^{-/-}$	N
EPSC1 (mean ± SEM, MWW)	1 ± 0	25	1 ± 0	27
EPSC 2 (mean ± SEM, STT)	0.77 ± 0.05	25	1.01± 0.05***	27
EPSC 3 (mean ± SEM, STT)	0.68 ± 0.07	25	1.08 ± 0.06***	27
EPSC4 (mean ± SEM, STT)	0.64 ± 0.08	25	1.03 ± 0.06***	27
EPSC 5 (mean ± SEM, STT)	0.59 ± 0.08	25	1 ± 0.06***	27
EPSC 6 (mean ± SEM, MWW)	0.58 ± 0.08	25	0.99 ± 0.06***	27
EPSC 7 (mean ± SEM, MWW)	0.55 ± 0.09	25	0.96 ± 0.06***	27
EPSC 8 (mean ± SEM, MWW)	0.54 ± 0.09	25	0.94 ± 0.06***	27
EPSC 9 (mean ± SEM, MWW)	0.55 ± 0.09	25	0.91 ± 0.07***	27
EPSC 10 (mean ± SEM, MWW)	0.53 ± 0.09	25	0.94 ± 0.09***	27

STT= Student's t-test; MWW = Mann-Whitney-Wilcoxon Test

Supplementary Table 9. EPSP rise time and τ_{decay} (see Fig.3a and Supplementary Fig. 5)

	**In the absence of NMDAR and $GABA_AR$ antagonists**					**In the presence of NMDAR and $GABA_AR$ antagonists**			
	WT	N	CKAMP44$^{-/-}$	N		WT	N	CKAMP44$^{-/-}$	N
10-90% rise time (ms) (median ± IQR, MWW)	1.52 [0.64-2.17]	22	1.2 [0.83-2.47]	21	10-90% rise time (ms) (median ± IQR, MWW)	0.98 [0.57-1.36]	20	0.75 [0.51-1.08]	20
τ_{decay} (ms) (mean ± SEM, STT)	19.9 ± 1.92	20	19.24 ± 1.68	22	τ_{decay} (ms) (median ± IQR, MWW)	17.36 [13.01-26.09]	20	15.54 [12.95-21.59]	20

STT= Student's t-test; MWW = Mann-Whitney-Wilcoxon Test

Supplementary Table 10. Passive and active electrophysiological properties of relay neurons (see Supplementary Fig. 6)

	WT	N	CKAMP44$^{-/-}$	N
Input resistance (MΩ)<br>(mean ± SEM, STT)	142 ± 7.93	21	126.11 ± 6.38	19
Resting potential (mV)<br>(median ± IQR, MWW)	-70<br>[(-72) -(-68.5)]	21	-70<br>[(-70) -(-69)]	19
AP threshold (mV)<br>(mean ± SEM, STT)	-41 ± 1.04	21	-41.67 ± 1.36	19
AP amplitude (mV)<br>(median ± IQR, MWW)	74.8<br>[71.5-78.7]	21	77.72<br>[75.55-80.45]	19
AP half amplitude duration (ms)<br>(median ± IQR, MWW)	0.92<br>[0.78-1.12]	21	0.82<br>[0.74-0.98]	19
AHP (mV) (mean ± SEM, STT)	6.6 ± 0.76	21	6.14 ± 0.71	19
Sag amplitude (mV) (mean ± SEM, STT)	3.41 ± 0.35	21	3.45 ± 0.42	19
Max frequency (Hz) (mean ± SEM, STT)	44.7 ± 1.75	21	49.16 ± 1.34	19
Early adaptation (%)<br>(median ± IQR, MWW)	238<br>[127-275]	21	215.39<br>[101.18-262.67]	19
Late adaptation (%)<br>(median ± IQR, MWW)	11<br>[7.34-17.9]	21	13.75<br>[10.02-18.01]	19

STT= Student's t-test; MWW = Mann-Whitney-Wilcoxon Test

Supplementary Table 11. Spike probability during 50 Hz stimulation (see Fig. 3b-e)

	**No stimulation before high frequency stimulus**				**40 stim (1 Hz) before high frequency stimulus**				**40 stim. (3.3 Hz) before high frequency stimulus**				**40 stim. (10 Hz) before high frequency stimulus**			
	WT	N	CK44	N	WT	N	CK44	N	WT	N	CK44	N	WT	N	CK44	N
1st stim.	0 ± 0	11	0 ± 0	10	0.004 ±0.004	27	0 ± 0	33	0 ± 0	20	0.01 ±0.01	32	0 ± 0	21	0 ± 0	27
2nd stim.	0.29 ±0.17	11	0.33 ±0.24	10	0.03 ±0.02	27	0.06 ±0.03	33	0.01 ±0.01	20	0.05 ±0.03	32	0 ± 0	21	0.01 ±0.01	27
3rd stim.	0.24 ±0.1	11	0.55 ±0.16	10	0.07 ±0.03	27	0.27 ±0.08	33	0.02 ±0.02	20	0.14 ±0.05	32	0 ± 0	21	0.02 ±0.02*	27
4th stim.	0.08 ±0.05	11	0.44 ±0.13**	10	0.07 ±0.04	27	0.29 ±0.08*	33	0.07 ±0.04	20	0.23 ±0.07	32	0 ± 0	21	0.05 ±0.03**	27
5th stim.	0 ±0	11	0.27 ±0.11**	10	0.02 ±0.01	27	0.27 ±0.07***	33	0.13 ±0.1	20	0.28 ±0.07	32	0.01 ±0.01	21	0.09 ±0.04*	27
6th stim.	0 ±0	11	0.32 ±0.18**	10	0.02 ±0.01	27	0.17 ±0.06**	33	0.04±0.04	20	0.20 ±0.05*	32	0.02 ±0.02	21	0.08 ±0.04*	27
7th stim.	0 ±0	11	0.36 ±0.20*	10	0.01 ±0.01	27	0.14 ±0.05**	33	0.01 ±0.01	20	0.11 ±0.03*	32	0.05±0.04	21	0.05 ± 0.02	27
8th stim.	0 ±0	11	0.4 ±0.21**	10	0.01 ±0.01	27	0.13 ±0.05**	33	0.01 ±0.01	20	0.08 ±0.03	32	0.02 ±0.02	21	0.03 ±0.0*	27
9th stim.	0 ±0	11	0.37 ±0.23*	10	0.004 ±0.004	27	0.1 ±0.05*	33	0 ± 0	20	0.05 ±0.03	32	0.02 ±0.02	21	0.03 ±0.02	27
10th stim.	0 ±0	11	0.51 ±0.24**	10	0.01 ±0.01	27	0.12 ±0.05*	33	0 ± 0	20	0.05 ±0.02*	32	0 ± 0	21	0.03 ±0.02*	27

For all: mean ± SEM; CK44 = CKAMP44$^{-/-}$; all with Mann-Whitney-Wilcoxon Test except the first, second and third stimulation data from" **No stimulation before high frequency stimulus**" with Student's t-test.

Supplementary Table 12. Firing rate during 50 Hz stimulation (see Fig. 3b-e right panel)

	**No stimulation before high frequency stimulus**				**40 stim (1 Hz) before high frequency stimulus**				**40 stim. (3.3 Hz) before high frequency stimulus**				**40 stim. (10 Hz) before high frequency stimulus**			
	WT	N	CK44	N	WT	N	CK44	N	WT	N	CK44	N	WT	N	CK44	N
Firing rate (Hz)	3.05 ±1.45	11	17.68 ±7.95	11	1.22 ±0.44	27	7.72 ±2**	33	1.3 ±0.77	20	6.02 ±1.81*	32	0.56 ±0.54	21	03.35 ±0.95*	27

For all: mean ± SEM; Mann-Whitney-Wilcoxon Test, CK44 = CKAMP44$^{-/-}$

Supplementary Table 13. Amplitude of 1st EPSP during low frequency stimulation (see Fig. 3b-e)

	WT	N	CKAMP44$^{-/-}$	N
1st EPSP without prior stimulation (mV)	14.36 ± 1.49	11	7.44 ± 0.58***	10
1st EPSP with 1 Hz prior stimulation (mV)	13.94 ± 1.21	27	7.54 ± 0.53***	33
1st EPSP with 3.3 Hz prior stimulation (mV)	13.52 ± 0.93	20	7.59 ± 0.53***	32
1st EPSP with 10 Hz prior stimulation (mV)	13.69 ± 1.24	22	7.89 ± 0.55***	40

For all: mean ± SEM; Mann-Whitney-Wilcoxon Test

Supplementary Table 14. EPSP amplitude reduction during low frequency stimulation (see Fig. 3b-e)

	WT	N	CKAMP44$^{-/-}$	N
EPSP reduction (%) (1 Hz)	29.11 ± 1.9	27	14.62 ± 4.47***	33
EPSP reduction (%) (3.3 Hz)	44.83 ± 1.96	20	28.2 ± 2.27***	32
EPSP reduction (%) (10 Hz)	63.1 ± 1.92	22	20.38 ± 6.33***	40

For all: mean ± SEM; Mann-Whitney-Wilcoxon Test

Supplementary Table 15. Firing rate with different number of stimuli at 25 Hz (see Fig. 4)

	WT	N	CKAMP44$^{-/-}$	N
Firing rate for 1 stimulus (Hz)	0 ± 0	50	0 ± 0	67
Firing rate for 2 stimuli (Hz)	3.61 ± 0.77	50	2.37 ± 0.66	67
Firing rate for 3 stimuli (Hz)	3.26 ± 0.66	50	4.03 ± 0.81	67
Firing rate for 4 stimuli (Hz)	2.59 ± 0.55	50	5.01 ± 0.88	67
Firing rate for 5 stimuli (Hz)	2.31 ± 0.49	50	6.24 ± 0.95*	67

For all: mean ± SEM; Mann-Whitney-Wilcoxon Test, CK44 = CKAMP44$^{-/-}$

Supplementary Table 16. Firing rate with different number of stimuli at 37.5 Hz (see Fig. 4)

	WT	N	CKAMP44$^{-/-}$	N
Firing rate for 1 stimulus (Hz)	0 ± 0	49	0 ± 0	28
Firing rate for 2 stimuli (Hz)	6.86 ± 1.62	49	3.89 ± 1.37	28
Firing rate for 3 stimuli (Hz)	7.95 ± 1.64	49	6.52 ± 1.9	28
Firing rate for 4 stimuli (Hz)	6.58 ± 1.28	49	8.95 ± 2.06	28
Firing rate for 5 stimuli (Hz)	5.57 ± 1.05	49	10.86 ± 2*	28
Firing rate for 6 stimuli (Hz)	5.1 ± 0.98	49	12.29 ± 2.03***	28
Firing rate for 7 stimuli (Hz)	4.81 ± 0.98	49	13.96 ± 2.02***	28

For all: mean ± SEM; Mann-Whitney-Wilcoxon Test, CK44 = CKAMP44$^{-/-}$

Supplementary Table 17. Firing rate with different number of stimuli at 50 Hz (see Fig. 4)

	WT	N	CKAMP44$^{-/-}$	N
Firing rate for 1 stimulus (Hz)	0 ± 0	45	0 ± 0	55
Firing rate for 2 stimuli (Hz)	11.82 ± 2.46	45	7.2± 2.1	55
Firing rate for 3 stimuli (Hz)	16.05 ± 2.84	45	13.64 ± 2.57	55
Firing rate for 4 stimuli (Hz)	13.39 ± 2.41	45	16.56 ± 2.4	55
Firing rate for 5 stimuli (Hz)	11.25 ± 2.11	45	17.96 ± 2.34*	55
Firing rate for 6 stimuli (Hz)	9.9 ± 1.98	45	19.65 ± 2.3***	55
Firing rate for 7 stimuli (Hz)	9.1 ± 1.91	45	21.65 ± 2.3***	55
Firing rate for 8 stimuli (Hz)	8.76 ± 1.9	45	24 ± 2.3***	55
Firing rate for 9 stimuli (Hz)	8.63 ± 1.94	45	26.66 ± 2.35***	55
Firing rate for 10 stimuli (Hz)	8.7 ± 2	45	29.83 ± 2.43***	55

For all: mean ± SEM; Mann-Whitney-Wilcoxon Test, CK44 = CKAMP44$^{-/-}$

Supplementary Table 18. Firing rate with different number of stimuli at suprathreshold stimulation strength (see Fig. 4)

	WT	N	CKAMP44$^{-/-}$	N
Firing rate for 1 stimulus (Hz) (mean ± SEM, STT)	51.61 ±4.46	31	50.91± 8.42	11
Firing rate for 2 stimuli (Hz) (mean ± SEM, STT)	44.52 ± 3.44	31	47.73 ± 4.91	11
Firing rate for 3 stimuli (Hz) (mean ± SEM, MWW)	38.39 ± 3.14	31	46.52± 3.87	11
Firing rate for 4 stimuli (Hz) (mean ± SEM, MWW	32.86 ± 2.95	31	45.34 ± 3.64*	11
Firing rate for 5 stimuli (Hz) (mean ± SEM, MWW)	28.93 ± 2.93	31	45.09 ± 3.45**	11
Firing rate for 6 stimuli (Hz) (mean ± SEM, STT)	25.96 ± 2.94	31	44.09 ± 3.38***	11
Firing rate for 7 stimuli (Hz) (mean ± SEM, STT)	23.88 ± 2.99	31	43.7 ± 3.44***	11
Firing rate for 8 stimuli (Hz) (mean ± SEM, STT)	22.26 ± 3.04	31	43.75 ± 3.66***	11
Firing rate for 9 stimuli (Hz) (mean ± SEM, STT)	21.25 ± 3.15	31	43.74 ± 3.9***	11
Firing rate for 10 stimuli (Hz) (mean ± SEM, STT)	20.2 ± 3.22	31	43.91 ± 4.29***	11

STT= Student's t-test; MWW = Mann-Whitney-Wilcoxon Test

Supplementary Table 19 PPR (30 ms inter-stimulus interval) in RG synapses of 2-month old mice (see Supplementary Fig. 7)

	WT	N	CKAMP44$^{-/-}$	N
PPR (median ± IQR, MWW)	0.4 [0.24-0.48]	12	0.77 [0.7-0.83] **	9

MWW = Mann-Whitney-Wilcoxon Test

Supplementary Table 20. ON- and OFF-responding neurons of in vivo recording (see Fig. 5)

	WT	N	CKAMP44$^{-/-}$	N
Number of recorded neurons per mouse (median ± IQR, MWW)	203.5 [164-279]	6	154.5 [134.2-231.8]	6
The proportion of ON-responding cells (mean ± SEM, STT)	0.40 ± 0.049	6	0.39 ± 0.046	6
The proportion of OFF-responding cells (mean ± SEM, STT)	0.22 ± 0.031	6	0.26 ± 0.039	6

MWW = Mann-Whitney-Wilcoxon Test; STT= Student's t-test

Supplementary Table 21 Peak firing rate and rate increase of ON- and OFF-responses (*in vivo*) (see Fig. 5 and Supplementary Fig. 8)

	Median ± IQR				Mean ± SEM			
	WT	N	CKAMP44$^{-/-}$	N	WT	N	CKAMP44$^{-/-}$	N
Peak firing rate of ON response (Hz)	14.42 [2.2-33.62]	542	15.89 [8.45-34.86] *	430	29.67 ±1.27	542	32.71 ±2.28	430
Peak firing rate of OFF-response (Hz)	11.23 [6.45-27.91]	309	15.08 [7.8-36.6] *	285	25.05 ±2.1	309	32.2 ±2.64	285
Rate increase of ON response (%)	319.6 [172.8-693.4]	542	370.4 [196.4-948.1] **	430	750.2 ±86.67	542	1039 ±120.5	430
Rate increase of OFF-response (%)	260 [163.5-731.5]	309	386.9 [190-957.1] **	285	731.5 ±114	309	957.1 ±95.48	285

Supplementary Table 22. mEPSCs of RGCs (see Supplementary Fig. 9)

	WT	N	CKAMP44$^{-/-}$	N
RGC mEPSC frequency (Hz)	0.46 [0.19-1.71]	24	0.46 [0.23-1.53]	29
RGC mEPSC amplitude (Hz)	11.02 [8.61-13.5]	24	10.26 [8.5-11.33]	29

For all: median ± IQR; Mann-Whitney-Wilcoxon Test

Supplementary Table 23. ERG recordings (see Supplementary Fig. 9b and c)

	WT	N	CKAMP44$^{-/-}$	N
fERG a-wave amplitude (µV) (mean ± SEM, STT)	28.17 ± 2.44	12	23.22 ± 3.08	12
fERG a-wave latency (ms) (mean ± SEM, STT)	16.67 ± 0.59	12	18.33 ± 0.81	12
fERG b-wave amplitude (µV) (mean ± SEM, STT)	122.78 ± 8.69	12	113.01 ± 13.35	12
fERG b-wave latency(ms) (median ± IQR, MMW)	24 [24-26]	12	26 [24-28.5]	12
pERG amplitude noise (µV) (median ± IQR, MMW)	2.37 [2.17-2.47]	6	2.3 [2.18-5]	6
pERG amplitude 0.2 cpd (µV) (median ± IQR, MMW)	8.51 [7.81-13.1]	6	8.9 [3.8-18.45]	6
pERG amplitude 0.1 cpd (µV) (median ± IQR, MMW)	4.17 [3.66-5.11]	6	5.29 [4.33-6.62]	6
PERG amplitude 0.05 cpd (µV) (median ± IQR, MMW)	3.93 [2.34-4.01]	6	5 [3-10.26]	6
pERG amplitude 0.025 cpd (µV) (median ± IQR, MMW)	5.4 [2.72-11.52]	6	5.03 [3.51-7.28]	6

STT= Student's t-test; MWW = Mann-Whitney-Wilcoxon Test
